# Exploration of the mechanisms and active compounds of *Scutellaria barbata* D. Don against influenza A (H1N1) virus

**DOI:** 10.3389/fmicb.2026.1945077

**Published:** 2026-10-01

**Authors:** Ying Yang, Junjing Shao, Lin Hou, Shihao Sun, Guihong Qi

**Affiliations:** 1 College of Basic Medicine, Jining Medical University, Jining, China; 2 Jining Key Laboratory of Basic and Clinical Transformation of Autoimmune Diseases, Jining Medical University, Jining, China; 3 Qingdao Academy of Chinese Medicinal Sciences, Shandong University of Traditional Chinese Medicine, Qingdao, China; 4 Beijing Life Science Academy, Beijing, China

**Keywords:** active compounds, antiviral, H1N1, mechanisms, *Scutellaria barbata* D. Don

## Abstract

**Introduction:**

*Scutellaria barbata* D. Don (*S. barbata*), a traditional Chinese medicinal herb widely used for its heat-clearing and detoxifying properties, has shown broad-spectrum antiviral potential. However, its anti-H1N1 activity and underlying mechanisms remain to be fully elucidated.

**Methods:**

In this study, the anti-H1N1 activity and underlying mechanisms of *S. barbata* extract were investigated using H1N1-infected MDCK cells and BALB/c mice. Transcriptomic analysis followed by mRNA and protein-level validation was performed to characterize its molecular effects, while UPLC-Q-Orbitrap-MS was used to identify blood-absorbed constituents. Potential active compounds were subsequently screened by integrating the absorbed constituents with network pharmacology and further validated *in vitro* and *in vivo*.

**Results:**

*S. barbata* extract markedly inhibited H1N1 replication in MDCK cells (IC_50_ = 42.87 μg/mL; CC_50_ > 500 μg/mL) and reduced viral burden and pulmonary inflammation in infected mice. Mechanistically, *S. barbata* treatment suppressed the Toll-like receptor, NF-κB, and chemokine signaling pathways, suggesting coordinated regulation of antiviral and inflammatory responses. Among the blood-absorbed constituents, baicalein and chrysoeriol were identified as key candidate active compounds and exhibited potent anti-H1N1 activity *in vitro* and *in vivo* (IC_50_ = 13.80 and 11.10 μM, respectively).

**Discussion:**

Collectively, these findings demonstrate that S. barbata exerts anti-H1N1 effects through a combination of direct inhibition of viral replication and modulation of excessive inflammatory responses, with baicalein and chrysoeriol representing important contributors to its antiviral activity.

## Introduction

Influenza A virus (IAV), a member of the genus Influenza virus within the family Orthomyxoviridae, is an enveloped, single-stranded, negative-sense RNA virus (Zeng et al., 2016). Since the 20th century, IAV has caused multiple global pandemics, at least three of which were closely associated with the H1N1 subtype (Krammer et al., 2018). The most recent pandemic, the 2009 H1N1 “swine flu” outbreak, resulted in at least 16,000 deaths worldwide. In addition to pandemics, IAV exhibits a pronounced seasonal epidemic pattern. Although seasonal strains generally display lower pathogenicity than emerging pandemic variants, they can still lead to severe lower respiratory tract infections and extensive lung injury. According to estimates by Iuliano et al. (2018) seasonal influenza causes approximately 291,243–645,832 deaths globally each year; among 92 surveyed countries, 9,243–105,690 annual deaths in children under 5 years of age were attributed to IAV infection (Iuliano et al., 2018). Furthermore, Wang et al. (2020) reported that in 2018, around 10 million children under 5 years old worldwide developed influenza-associated acute lower respiratory tract infections, resulting in approximately 870,000 hospitalizations and 35,000 deaths.

Following IAV infection, the host’s innate immune system is rapidly activated. Infected host cells recognize viral pathogen-associated molecular patterns (PAMPs) through various pattern recognition receptors (PRRs), thereby initiating innate immune signaling pathways. Once viral RNA enters the cytoplasm, it activates three principal intracellular immune pathways: the retinoic acid-inducible gene I (RIG-I) pathway, the toll-like receptor (TLR) pathway, and the inflammasome pathway, all of which contribute to antiviral innate immunity. During the early phase of IAV infection, immune responses occur primarily in infected airway and alveolar epithelial cells. Viral RNA activates the TLR signaling pathway, inducing the expression of key signaling molecules that promote the activation of interferon regulatory factors (IRFs) and enhance the transcription of type I interferons (IFN-α/β). Moreover, activation of this pathway can further stimulate the nuclear factor-κB (NF-κB) signaling cascade, leading to the expression of pro-inflammatory cytokines and chemokines. The combined effects of type I interferons, pro-inflammatory cytokines, and the activation and differentiation of cytotoxic T cells ultimately contribute to viral clearance (Jagger et al., 2012).

Although traditional Chinese medicine (TCM) does not include the concept of “virus,” antiviral therapy within TCM dates back to the Han dynasty, notably to Zhang Zhongjing’s Treatise on Cold Damage and Miscellaneous Diseases. According to TCM theory, IAV infection belongs to the category of “Warm Diseases” and is closely related to pulmonary disorders caused by “external heat pathogens” (Cao et al., 2009; Wise et al., 2009). TCM herbal medicines are characterized by multi-component, multi-target, and multi-pathway mechanisms. Their active extracts can inhibit viral replication, disrupt viral structure, regulate immune balance, and maintain systemic homeostasis, reflecting the holistic regulatory principles of TCM (Zhang et al., 2013).

In our previous screening of herbal medicines with anti-H1N1 activity, the aqueous extract of *Scutellaria barbata* D. Don (*S. barbata*) exhibited significant antiviral potential. *S. barbata*, a member of the Lamiaceae family and the genus *S. barbata* is the dried whole herb traditionally used for its heat-clearing, detoxifying, blood-stasis-resolving, hemostatic, diuretic, and anti-edematous properties. Modern pharmacological studies have demonstrated that heat-clearing and detoxifying herbs generally possess strong antiviral potential. Extracts of *S. barbata* have been shown to alleviate SARS-CoV-2–induced acute lung injury by inhibiting viral replication and modulating immune responses (Ran et al., 2025); its combined extract with *Hedyotis diffusa* suppresses hepatitis B virus–related hepatocellular carcinoma growth by regulating circular RNA (circRNA) expression (Yang et al., 2021); diterpenoids isolated from *S. barbata* display inhibitory activity against Epstein–Barr virus lytic replication (Wu et al., 2015); and its total flavonoid fraction exhibits anti-parainfluenza virus activity (Shan-Shan et al., 2009).

Based on these findings, the present study established H1N1-infected MDCK cell and BALB/c mouse models to systematically evaluate the inhibitory effects of *S. barbata* extract against IAV (H1N1 subtype). The results demonstrated that *S. barbata* exerts anti-H1N1 activity by directly suppressing viral replication and modulating anti-inflammatory and immune-related signaling pathways. Baicalein and chrysoeriol were confirmed to be key constituents responsible for the antiviral activity of *S. barbata*.

## Materials and methods

### Materials

Chrysoeriol (C861251) was obtained from Macklin (Shanghai, China), and baicalein (B20571) was purchased from Yuan Ye Biotechnology (Shanghai, China). Dulbecco’s Modified Eagle Medium (DMEM, 812207), fetal bovine serum (FBS, 10270-106), 0.25% trypsin–EDTA (2323363), phosphate-buffered saline (PBS, BYHC1713), and Opti-MEM medium (31985070) were all supplied by Gibco (Massachusetts, USA). MDCK cells (as a host of H1N1) and IA V [A/Puerto Rico/8/1934(H1N1)] were provided by the Shandong Academy of Medical Sciences (Jinan, China). Enzyme-linked immunosorbent assay (ELISA) kits for TNF-α (JL10484), IL-1β (JL18442), IL-6 (JL20268), IL-2 (JL20256), IFN-γ (JL10967), and IL-4 (JL20266) were obtained from Shanghai Jianglai Biotechnology Co., Ltd., (Shanghai, China). The anti-influenza A virus nucleoprotein antibody (ab20343) was purchased from Abcam (Cambridge, UK). Fluorochrome-conjugated antibodies, including FITC anti-mouse CD4 (116004), PE anti-mouse IL-4 (504104), APC anti-mouse IFN-γ (505809), and APC anti-mouse CD8b (Ly-3) (126614), were supplied by BioLegend (California, USA).

### Sample preparation

The extract of *S. barbata* was prepared using a water extraction method. Briefly, 100 g of dried herb powder was mixed with 1,000 mL of deionized water and subjected to ultrasonic-assisted extraction at 250 W for 1 h. The resulting extract was filtered, concentrated under reduced pressure, and lyophilized to yield a dry powder. For subsequent experiments, the lyophilized extract was reconstituted according to its experimental application: dissolved in Opti-MEM medium for *in vitro* cellular assays and in PBS buffer for *in vivo* animal studies. Both solutions were further diluted to the required working concentrations for pharmacodynamic evaluation.

### Study on anti-H1N1 activity of *S. barbata in vitro*

MDCK cells were treated with varying concentrations of *S. barbata* extract and oseltamivir phosphate (OP) for 48 h. Cytotoxicity was assessed using the MTT assay to determine the 50% cytotoxic concentration (CC_50_) for both agents. The highest tested concentration that maintained cell viability above 90% was defined as the non-cytotoxic concentration for subsequent experiments. For viral infection studies, MDCK cells were seeded in 96-well plates at a density of 1 × 10^5^ cells/mL (100 μL per well) and cultured for 24 h at 37 °C with 5% CO_2_. Following incubation, the culture medium was removed, and cell monolayers were washed twice with PBS. Cells were then infected with H1N1 virus (100 TCID_50_/well) in Opti-MEM medium supplemented with 1,000 U/mL penicillin, 100 μg/mL streptomycin, and 2 μg/mL TPCK-trypsin, while simultaneously being treated with different concentrations of either *S. barbata* extract or OP. After 48 h of incubation, cell viability was measured using the MTT assay. The half-maximal inhibitory concentration (IC_50_) values for *S. barbata* and OP against H1N1 viral infection were calculated according to the Reed-Muench method (Chen et al., 2010; Geng et al., 2019).

MDCK cells were seeded in 12-well plates at a density of 8 × 10^4^ cells per well and cultured until full confluence was reached. The cell monolayers were then washed twice with PBS and replenished with fresh Opti-MEM medium. Subsequently, the cells were treated with either *S. barbata* extract or OP, followed by immediate infection with H1N1 virus at a dose of 100 TCID_50_ per well. The infected cells were maintained at 37 °C under 5% CO_2_ for 24 h. To quantify viral gene expression, cells subjected to the above treatment were harvested after three freeze-thaw cycles, and the supernatant was collected following centrifugation. The mRNA expression level of the H1N1 NP gene in the supernatant was detected by Quantitative real-time PCR (qRT-PCR). For the analysis of viral protein expression, MDCK cells grown on coverslips in 12-well plates were treated and infected under identical conditions as described above. After 24 h of infection, the coverslips were collected, and the expression level of H1N1 NP protein in MDCK cells was determined by immunofluorescence assay.

### Study on the anti-H1N1 activity of *S. barbata in vivo*

All animal experiments were reviewed and approved by the Laboratory Animal Care and Use Committee of Shandong University of Traditional Chinese Medicine (approval no. SDUTCM20220415022). Specific pathogen-free (SPF) female BALB/c mice (6 weeks old, 15–18 g) were purchased from Beijing Vital River Laboratory Animal Technology Co., Ltd., [Animal production license no. SCXK (Jing) 2021-0006; animal use license no. SYXK (Lu) 2017-0022]. Mice were housed in an individually ventilated cage (IVC) system under controlled environmental conditions (temperature 20 °C–24 °C) with free access to standard chow and water, and were acclimatized for 3 days prior to experimentation.

The mice were randomly divided into five groups: control group, model group, OP group (20 mg⋅kg^–1^), *S. barbata* (390 mg⋅kg^–1^) group, and *S. barbata* (78 mg⋅kg^–1^) group. Following anesthesia with a small-animal anesthesia system (RWD Life Science, China), mice were intranasally inoculated with 20 μL 0.8 LD_50_ H1N1 virus. Two hours post-infection, drug administration was initiated by oral gavage once daily for five consecutive days. At the end of the experiment, peripheral blood samples were collected from the mice under light anesthesia. Then, mice were euthanized by overdose of isoflurane anesthesia (5% isoflurane delivered at 2 L/min for 10 min), and death was confirmed before tissue collection. Lung, spleen, and thymus tissues were immediately collected for subsequent analyses.

### QRT-PCR

Viral RNA was extracted using the TIANamp Virus RNA Kit (Tiangen, China), and total RNA from lung tissues was isolated using the RNA Easy Fast Tissue/Cell Kit (Tiangen, China). Reverse transcription and quantitative PCR amplification were performed using the BeyoFast™ SYBR Green One-Step qRT-PCR Kit (Beyotime, China). The specific primer sequences are listed in Table 1. The thermal cycling conditions were as follows: reverse transcription at 50 °C for 15 min; initial denaturation at 95 °C for 2 min; followed by 40 cycles of denaturation at 95 °C for 15 s and annealing/extension at 60 °C for 30 s. GAPDH was used as the internal reference gene, and the relative expression levels of the viral NP gene were calculated using the 2^–ΔΔ*Ct*^ method.

**TABLE 1:** Primer sequences for quantitative reverse transcription-polymerase chain reaction.

Gene	Direction	Nucleotide sequence (5′→ 3)
CCL11	FOR	GCTCACGGTCACTTCCTT
CCL11	REV	GCGACTGGTGCTGATATTC
CCL12	FOR	CTACCACCATCAGTCCTCAGGTA
CCL12	REV	GGCTGCTTGTGATTCTCCTGTAG
CCL2	FOR	GGTGTCCCAAAGAAGCTGTAGTT
CCL2	REV	AGCTTCAGATTTACGGGTCAACT
CCL3	FOR	CAGACACCAGAAGGATACAA
CCL3	REV	ACAGAGAAGAACAGCAAGG
CCL4	FOR	TCACTGAGAAGCGTCCTT
CCL4	REV	GTCATATCCACAATAGCAGAG
CCL6	FOR	GAGGAAGTTCAGGTAGTTGT
CCL6	REV	CTTGGAGTATAGGAAGGAGTT
CCL7	FOR	TCACTCTCTTTCTCCACCATGAG
CCL7	REV	CAGACTTCCATGCCCTTCTTTGT
CCL8	FOR	CAGATAAGGCTCCAGTCAC
CCL8	REV	AGGATCTCCATGTACTCACT
CCR1	FOR	GTTGGGACCTTGAACCTTGAATT
CCR1	REV	GGGTAGGCTTCTGTGAAATCTGA
CCR5	FOR	TTCCTGCTCACACTACCA
CCR5	REV	CCTCTTCTTCTCATTCCTACA
CD14	FOR	TACAACAGGCTGGATAGGA
CD14	REV	CCACTGAACCATCTTGACT
CD80	FOR	CTCGCAACCACACCATTA
CD80	REV	CCTCAGAATCAGCAGAACTA
CXCL1	FOR	CTGCACCCAAACCGAAGTCATAG
CXCL1	REV	CCATCGGTGCAATCTATCTTCTTTC
CXCL13	FOR	GCGAGATGGACTTCAGTTA
CXCL13	REV	GAATGACACCAGATGAATAGG
CXCL16	FOR	CCAGCAGACAGACAGATG
CXCL16	REV	GGAAGAACGCAAGAGACA
CXCL2	FOR	CCTCAACGGAAGAACCAA
CXCL2	REV	TACAGTGAATTAGCGAATGC
CXCL3	FOR	TTTGAGAACATCCAGAGCTTGAC
CXCL3	REV	AGCTTGAGGGTTGAGACAAACTT
CXCL9	FOR	CTACTGCCTCCTGAATGC
CXCL9	REV	CGCTGAAGATGTCTGAAGA
GAPDH	FOR	GAGTCCACTGGCGTCTTCAC
GAPDH	REV	TGCTGATGATCTTGAGGCTGTT
HA (H1N1)	FOR	TCCGTCCATTCAATCCAGAGG
HA (H1N1)	REV	GATCCGCTGCATAGCCTGAT
IL-6	FOR	TTCCATCCAGTTGCCTTCTTG
IL-6	REV	AATTAAGCCTCCGACTTGTGAA
M2 (H1N1)	FOR	GCAACGGTTCAAGTGATCCTC
M2 (H1N1)	REV	ACCATCGTCAGCATCCACAG
Myd88	FOR	ACCTGTGTCTGGTCCATTGC
Myd88	REV	GGAATCAGTCGCTTCTGTTGG
NA (H1N1)	FOR	GGCTGGCTAACAATCGGAATT
NA (H1N1)	REV	ACACAGGCACATTCAGACTCT
NP (H1N1)	FOR	GTATGGACCTGCCGTAGC
NP (H1N1)	REV	GCTTCCACCAGGGTTCTC
RelA	FOR	CAACAGCACAGACCCAGGAG
RelA	REV	TGTCACCAGGCGAGTTATAGC
TLR4	FOR	CCATTGCTGCCAACATCATCC
TLR4	REV	GCGATACAATTCCACCTGCTG
TLR7	FOR	GCTACCAGGACAGCCAGTTC
TLR7	REV	TCCAGGAGCCTCTGATGAGAC
TLR8	FOR	GAACATCACTCAGGAAGACT
TLR8	REV	TCGGAACACATAGCCTCT
Traf6	FOR	GAATCACTTGGCACGACACTTG
Traf6	REV	TACTAGGCGACTCTCCAACTGT

### Immunofluorescence analysis

MDCK cells were seeded onto glass coverslips placed in 12-well plates (Beyotime, China) and subjected to viral infection and drug treatment according to the experimental design. Following treatment, the cells were washed three times with pre-cooled PBS, fixed with 4% paraformaldehyde at room temperature for 20 min, and permeabilized with 0.1% Triton X-100 for 15 min. After blocking with 2% BSA for 30 min, cells were incubated overnight at 4 °C with an anti-influenza A virus NP monoclonal antibody, followed by incubation with AF488-conjugated goat anti-mouse IgG (Bioss, China) at room temperature for 1 h in the dark. Nuclei were counterstained with DAPI (Beyotime, China) for 5 min. Fluorescence images were captured using an Olympus fluorescence microscope (Olympus, Japan).

### Enzyme-linked immunosorbent assay (ELISA)

The concentrations of TNF-α, IL-1β, IL-6, IL-2, IFN-γ, and IL-4 in lung tissue homogenates were quantified using commercial ELISA kits (Jianglai, China) according to the manufacturer’s instructions.

### Histopathological examination

Lung tissues were fixed in 4% paraformaldehyde for 48 h, embedded in paraffin, and sectioned at 4 μm thickness. Hematoxylin–eosin (H&E) and immunohistochemical (IHC) staining were performed. For H&E staining, sections were stained with hematoxylin and eosin to assess inflammatory cell infiltration and pathological alterations. For IHC staining, sections underwent antigen retrieval and 5% BSA blocking, followed by incubation with primary antibodies against TLR4 and RelA at 4 °C overnight, and then with HRP-conjugated secondary antibodies at room temperature for 30 min. Color development was performed using DAB, nuclei were counterstained with hematoxylin, and slides were mounted with neutral resin for microscopic observation.

### Transcriptomic analysis

Total RNA extraction and RNA-seq of lung tissues were conducted by BGI Tech Solutions Co., Ltd., (Wuhan, China). Differentially expressed genes (DEGs) were identified with |log_2_(fold change)| > 1 and adjusted *p* < 0.05. Overlapping DEGs between the control and model groups, and between the treatment and model groups, were subjected to hierarchical clustering, Gene Ontology (GO) annotation, and KEGG pathway enrichment analysis.

### Flow cytometry analysis

Spleens were gently ground through a 200-mesh stainless steel sieve to obtain single-cell suspensions. After red blood cell lysis, cells were resuspended in flow cytometry buffer (PBS containing 1% FBS). For Th1/Th2 analysis, cells were stimulated with phorbol 12-myristate 13-acetate (50 ng/mL), ionomycin (1 μg/mL), and brefeldin A (10 μg/mL) at 37 °C for 4 h, followed by surface staining with CD4-FITC, fixation/permeabilization, and intracellular staining with IFN-γ-APC and IL-4-PE. For T cell subset analysis, cells were stained with PE-CD3, APC-CD4, and FITC-CD8 antibodies. Data were acquired on a BD flow cytometer (BD Biosciences, USA) and analyzed using FlowJo software.

### Analysis of blood-absorbed components

Twelve BALB/c mice were randomly divided into model and treatment groups. H1N1 infection was established as described previously. The treatment group received *S. barbata* extract (3.9 g⋅kg^–1^, ten times the effective dose) via oral gavage, while the model group received an equal volume of PBS for six consecutive days. Peripheral blood samples were collected from the orbital venous plexus of mice under light anesthesia at 10 min, 0.5, 1, 2, and 4 h after the final administration. Blood samples were collected into heparinized tubes and centrifuged at 3,000 rpm for 10 min at 4 °C to obtain plasma. Each 200 μL plasma sample was mixed with 20 μL of 20% ascorbic acid and 800 μL of methanol, vortexed, and centrifuged at 14,000 rpm for 10 min. The supernatant was analyzed using UPLC-Q-Orbitrap-MS (Thermo Fisher Scientific, USA) with an Ultimate 3,000 UHPLC system coupled to a Q Exactive™ quadrupole-Orbitrap mass spectrometer. Chromatographic separation was achieved at 45 °C using a flow rate of 0.35 mL/min and an injection volume of 5 μL. The mobile phases consisted of 0.1% formic acid in water (A) and acetonitrile (B) with the following gradient: 0–2 min (95% A), 2–4 min (95%–70% A), 4–8 min (70%–50% A), 8–10 min (50%–20% A), 10–14 min (20%–0% A), and 14–15 min (0% A). The MS parameters were as follows: negative ion mode spray voltage, 3200 V; positive ion mode, 3,800 V; capillary temperature, 320 °C; full-scan resolution, 60,000; MS/MS resolution, 15,000; scan range, m/z 100–1,500; stepped collision energy, 10/20/40 eV. Data were processed using Xcalibur 3.0 software. The structures of the compounds were determined through analysis of fragmentation patterns, and confirmed by comparison with both reference standards and database information (MassBank, FooDB), as well as supporting literature.

### Network pharmacology analysis of potential active components

Potential targets of blood-absorbed compounds were identified using the PubChem and SwissTargetPrediction databases (Daina et al., 2019; Hao et al., 2013). In PubChem, the search parameters were set to “active” for activity and “9606” for taxids. In SwissTargetPrediction, the filter “homospines” and a probability threshold of >0.05 were applied. The combined target lists were standardized to gene symbols using the UniProt database (Piñero et al., 2021; Stelzer et al., 2016). DEGs (*n* = 1,907) that significantly differed between both the treatment vs. model and control vs. model groups were defined as disease-associated targets. The intersection between drug and disease targets was identified using Venny 2.1. KEGG enrichment analysis was performed on overlapping targets using the Metascape platform. The compound–target–pathway–disease interaction network was visualized using Cytoscape 3.6.0.

### Validation of monomer compound activity

Standard compounds of chrysoeriol and baicalein were accurately weighed, dissolved in DMSO, and diluted with Opti-MEM medium containing 2 μg/mL TPCK-treated trypsin. The antiviral and cytotoxicity assays were performed following the same procedures described for in “*Study on anti-H1N1 activity of S. barbata in vitro.*” Mice were randomly assigned to the control, model, OP (20 mg/kg), chrysoeriol (20 or 4 mg/kg), and baicalein (20 or 4 mg/kg) groups. After a 3-days acclimation, mice were anesthetized with isoflurane using a small animal anesthesia system, followed by intranasal inoculation with 20 μL of H1N1 virus suspension containing 0.8 LD_50_. Two hours post-infection, the mice received oral administration of 0.2 mL of the respective treatments. Organ indices, histopathological evaluation, and pulmonary viral load determination were performed as described in the “*Study on the anti-H1N1 activity of S. barbata in vivo*” section.

## Results

### *In vitro* antiviral activity of *S. barbata*

*In vitro* assays demonstrated that *S. barbata* and OP exhibited dose-dependent inhibitory effects against H1N1 infection in MDCK cells (Figures 1A, B). The calculated IC_50_ values of *S. barbata* and OP were 42.87 and 7.02 μg⋅mL^–1^, respectively. Both the viral load (Figure 1C) and NP fluorescence intensity (green signal) were significantly lower in the *S. barbata* treatment group compared with the model group (Figure 1D), indicating that *S. barbata* markedly inhibited viral replication within H1N1-infected MDCK cells.

**FIGURE 1:**
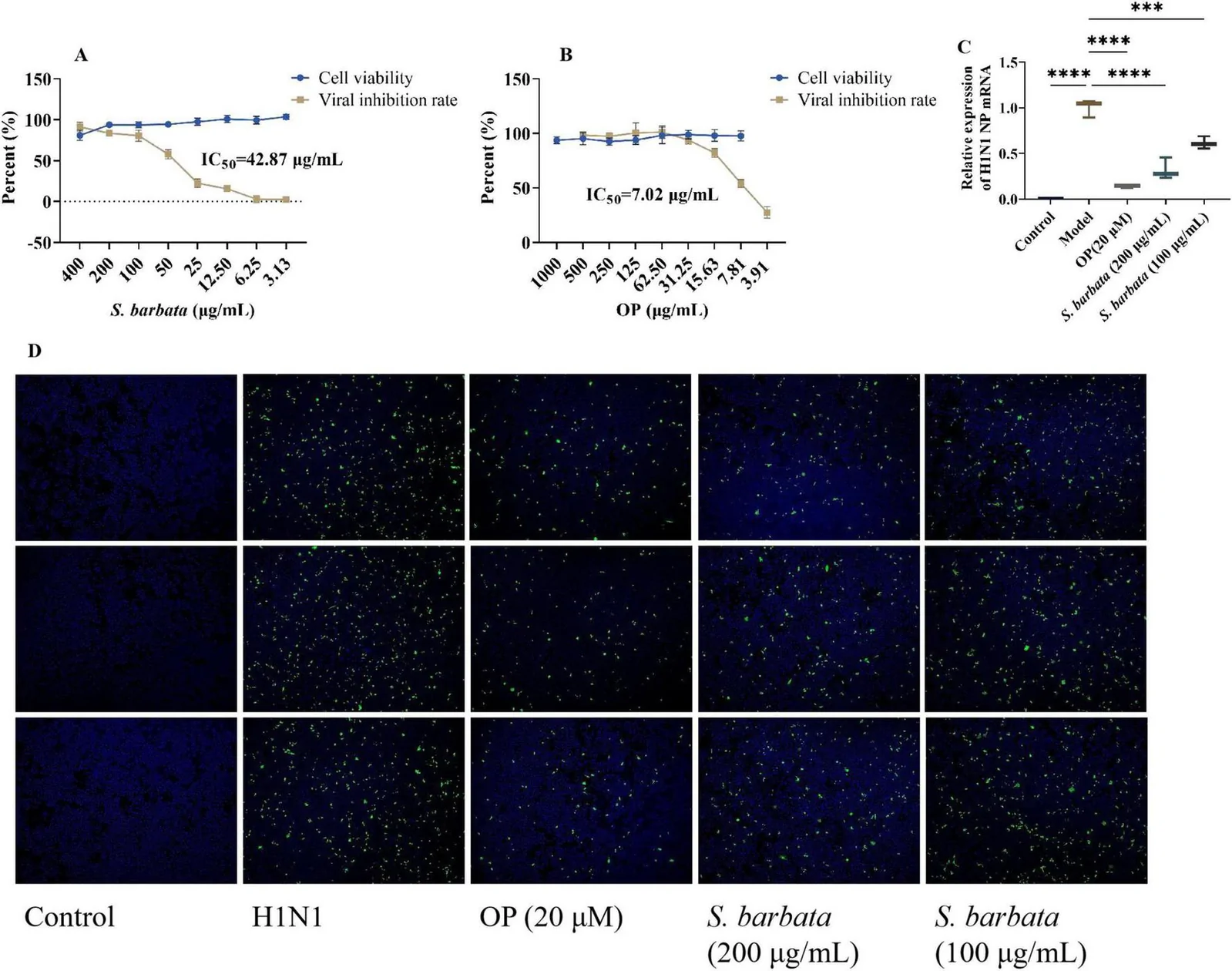
*In vitro* anti-IAV H1N1 assays. Antiviral activity of *S. barbata* **(A)** and OP **(B)** against H1N1 *in vitro*; viral load in cell supernatant **(C)** and NP protein expression in MDCK cells **(D)** (*n* = 3). ***P < 0.001 and ****P < 0.0001 indicate significant differences compared with the model group.

### *In vivo* antiviral efficacy of *S. barbata*

Following intranasal infection with 0.8 LD_50_ of H1N1 virus, mice in the model group exhibited progressive body weight loss and clinical symptoms such as ruffled fur and reduced activity starting on day 4 post-infection. These effects were notably alleviated in the *S. barbata* and OP treatment groups (Figure 2A). On day 6 post-infection, mice were euthanized, and the lung, spleen, and thymus were collected and weighed to calculate organ indices. Compared with the control group, the lung index of infected mice significantly increased (Figure 2B), while the thymus (Figure 2C), and spleen indices (Figure 2D) decreased. *S. barbata* treatment significantly reduced the lung index while elevating spleen and thymus indices relative to the model group. qRT-PCR analysis revealed that the mRNA expression levels of four H1N1 genes–Hemagglutinin (HA) (Figure 2E), neuraminidase (NA) (Figure 2F), M2 (Figure 2G) and NP (Figure 2H)–were markedly decreased in lung tissues from *S. barbata*-treated mice compared with the model group, suggesting suppression of viral replication *in vivo*. Consistent with these findings, immunofluorescence staining showed that NP fluorescence intensity in lung tissues was markedly reduced in the *S. barbata* group (Figure 2I), further confirming a decrease in pulmonary viral load.

**FIGURE 2:**
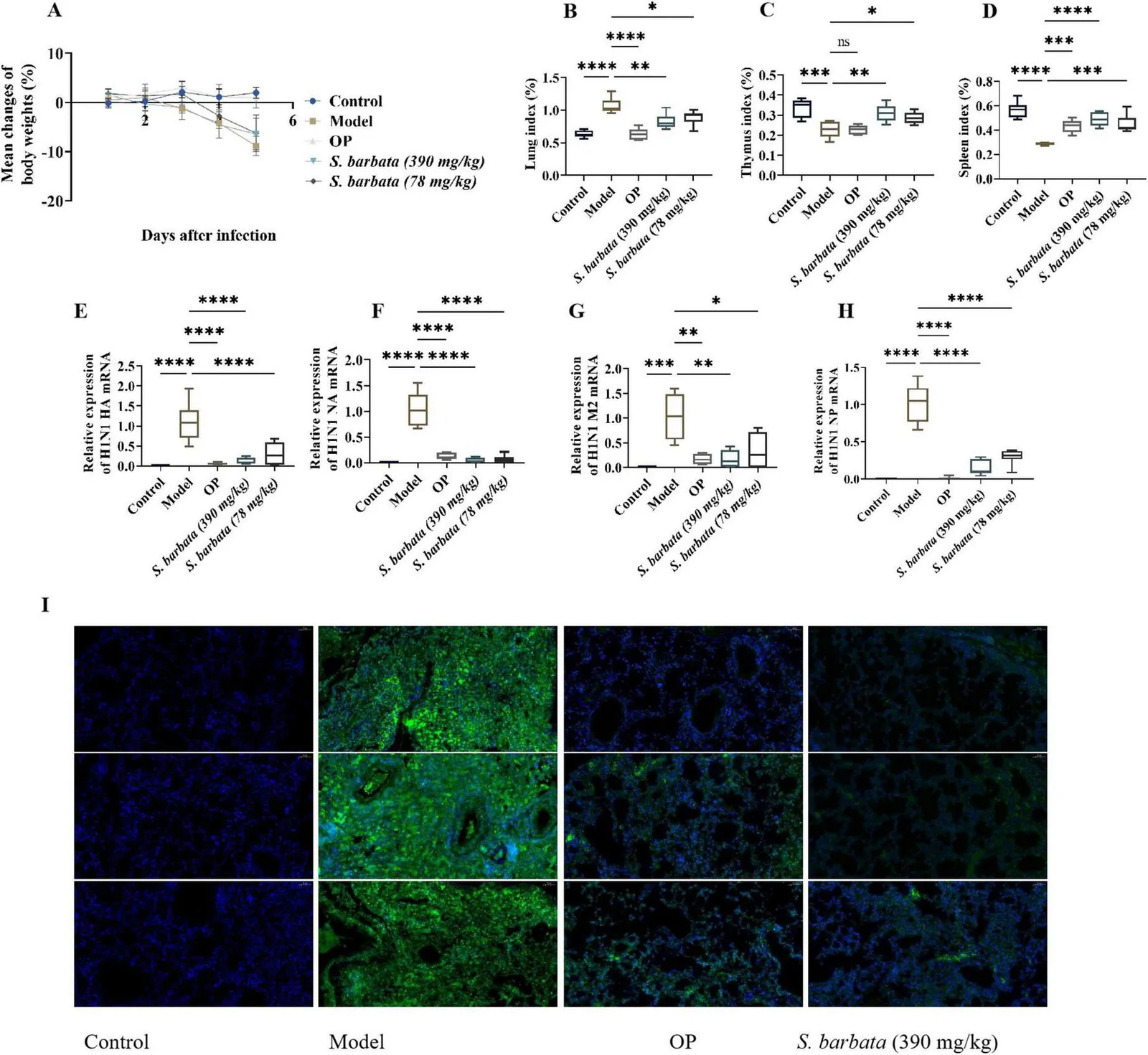
*In vivo* anti-IAV H1N1 assays (*n* = 6). Mean changes of body weight **(A)**, lung index **(B)**, thymus index **(C)**, spleen index **(D)**. The mRNA levels of HA **(E)**, NA **(F)**, M2 **(G)**, NP **(H)** in mouse lung tissues. NP protein expression in lung tissues **(I)**. *P < 0.05, **P < 0.01, ***P < 0.001, and ****P < 0.0001 indicate significant differences compared with the model group; ns indicates no significant difference.

### Effects of *S. barbata* on lung inflammation and cytokine expression

Based on the analysis of organ indices, *S. barbata* significantly reduced the lung index in H1N1-infected mice. As the lung index reflects the extent of pulmonary inflammatory infiltration, this finding suggests that *S. barbata* may alleviate H1N1-induced inflammatory injury in lung tissues. Therefore, we further investigated the inhibitory effect of *S. barbata* on H1N1-induced inflammatory responses. In the model group, lung tissues showed thickened alveolar septa, narrowed alveolar spaces, focal necrosis of alveolar epithelial cells, extensive inflammatory cell infiltration, and marked vascular congestion. Treatment with *S. barbata* alleviated alveolar septal thickening and inflammatory infiltration (Figure 3A). In the OP group, alveolar injury and inflammatory infiltration were also improved, though mild vascular congestion was still observed.

**FIGURE 3:**
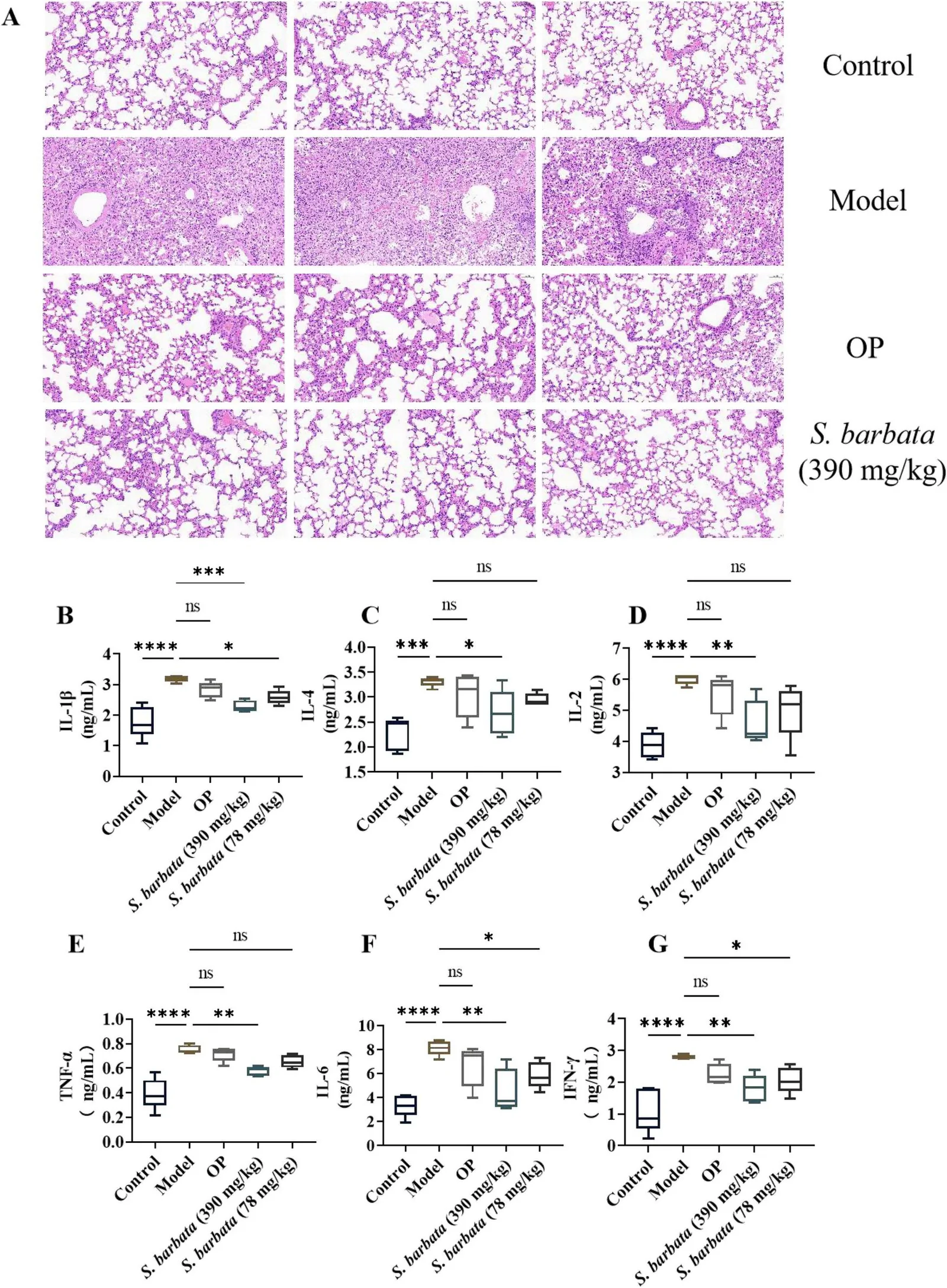
The effects of *S. barbata* on levels of inflammatory in lung tissues and lung lesions were investigated. Histopathological examination **(A)** (*n* = 3). The levels of IL-1β **(B)**, IL-4 **(C)**, IL-2 **(D)**, TNF-α **(E)**, IL-6 **(F)**, IFN-γ **(G)** in lung tissues (*n* = 6). *P < 0.05, **P < 0.01, ***P < 0.001, and ****P < 0.0001 indicate significant differences compared with the model group; ns indicates no significant difference.

Compared with the control group, H1N1-infected mice exhibited significantly elevated levels of IL-6, IL-2, TNF-α, IL-4, IFN-γ, and IL-1β, which are endogenous pyrogens that activate M1-type macrophages and promote the clearance of infected cells (Dinarello, 1999). However, excessive cytokine production can lead to immunopathological damage. Compared with the model group, *S. barbata* (390 mg⋅kg^–1^) treatment significantly reduced the levels of all six cytokines in lung tissues. The lower dose (78 mg⋅kg^–1^) significantly decreased IL-6, IFN-γ, and IL-1β levels, while IL-2, TNF-α, and IL-4 levels showed a downward trend without statistical significance. These findings suggest that high-dose *S. barbata* effectively attenuates H1N1-induced inflammatory injury in the lungs (Figures 3B–G).

### Effects of *S. barbata* on immune regulation

Analysis of organ indices also revealed that *S. barbata* markedly increased spleen and thymus indices in H1N1-infected mice, suggesting an immunomodulatory effect. Flow cytometry was used to quantify the proportions of CD4^+^ and CD8^+^ T cells in splenocytes. Compared with the control group, the percentage of CD4^+^ T cells in the peripheral blood of mice in the model group was significantly increased (Figure 4A), while the percentage of CD8^+^ T cells showed no significant change (Figure 4B). Treatment with *S. barbata* markedly reduced the proportion of CD4^+^ T cells compared with the model group, whereas the percentage of CD8^+^ T cells remained unchanged. Previous studies have indicated that imbalance in the CD4^+^/CD8^+^ T cell ratio may correlate with the severity of H1N1 pneumonia (Kim et al., 2011). Consistent with this, our results showed that the CD4^+^/CD8^+^ ratio was significantly elevated in the model group relative to controls, but was restored toward control levels by *S. barbata* and OP treatment, suggesting that *S. barbata* can rebalance CD4^+^/CD8^+^ T cell ratios disrupted by H1N1 infection (Figure 4C).

**FIGURE 4:**
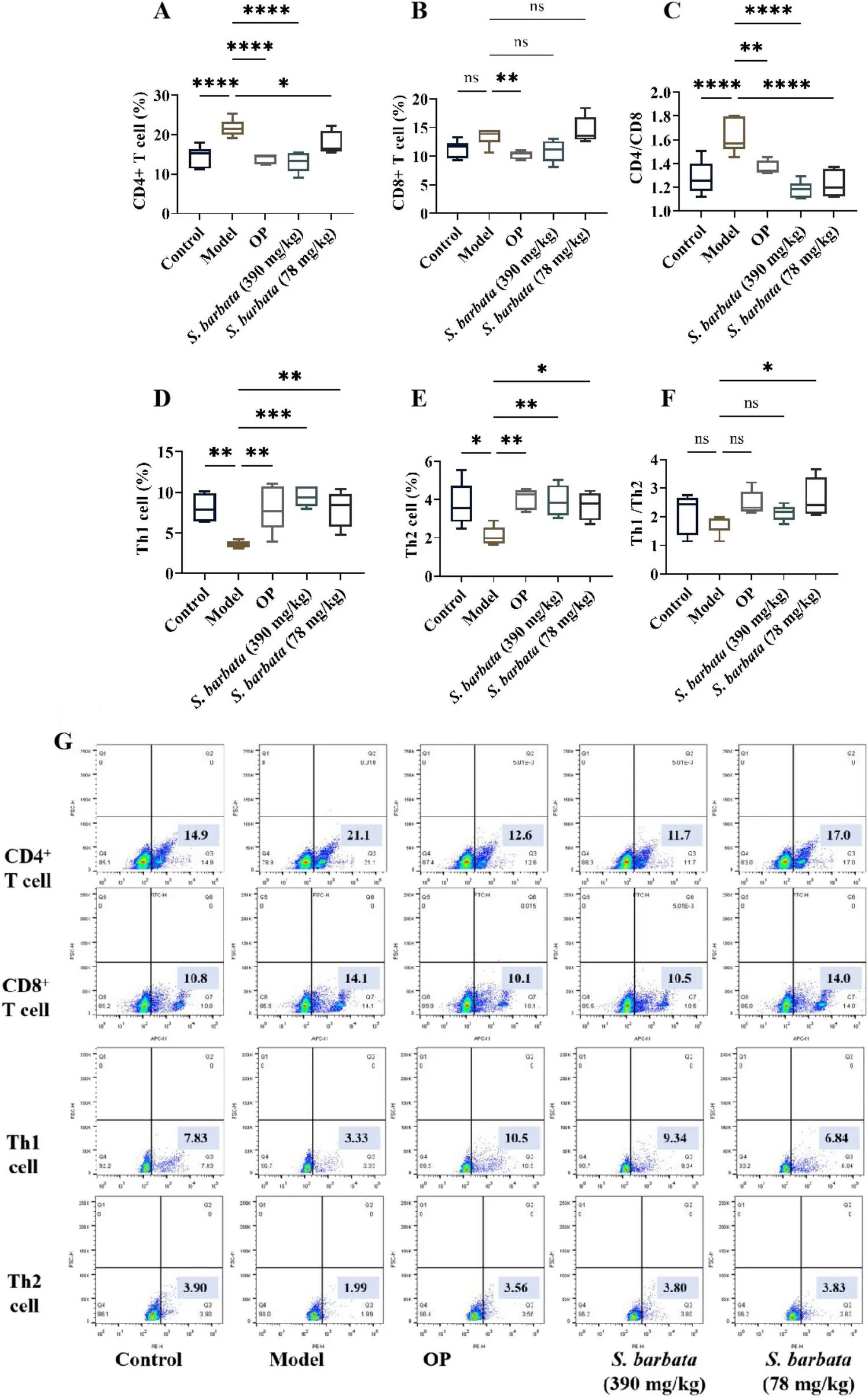
Effects of *S. barbara* on CD4/CD8 and Th1/Th2 balance. The percentage of CD4+ **(A)** and CD8+ **(B)** T cells; the ratio of CD4+ to CD8+ T cells **(C)**. The percentage of Th1 **(D)** and Th2 **(E)** cells; the ratio of Th1 to Th2 cells **(F)**. The percentage of CD4, CD8, Th1 and Th2 cells in the spleen (*n* = 5) **(G)**. *P < 0.05, **P < 0.01, ***P < 0.001, and ****P < 0.0001 indicate significant differences compared with the model group; ns indicates no significant difference.

To further elucidate its immunomodulatory effects, we examined the differentiation of CD4^+^ T cell subsets, including Th1 (CD4^+^IFN-γ^+^) and Th2 (CD4^+^IL-4^+^) cells. Compared with the control group, both Th1 and Th2 cell proportions were significantly reduced in H1N1-infected mice (Figures 4D, E). Treatment with *S. barbata* significantly restored the percentages of both subsets. However, the Th1/Th2 (CD4^+^IFN-γ^+^/CD4^+^IL-4^+^) ratio showed no significant difference among groups, likely due to parallel increases or decreases in the two subsets (Figure 4F). Representative flow cytometry plots and the corresponding quantitative analysis are presented in Figure 4G.

### Transcriptomic analysis

Differentially expressed genes were identified using the criteria of |log_2_FC| > 1 and *P* < 0.05, yielding a total of 3,084 DEGs between the *S. barbata*-treated and model groups. Among these, 1,369 genes were significantly upregulated in the model group relative to the treatment group (indicating downregulation upon *S. barbata* treatment), while 1,715 genes were significantly downregulated in the model group (indicating upregulation by treatment) (Figure 5A). Further comparison revealed that 1,916 of these DEGs also showed significant expression differences between the control and model groups. Specifically, 1,018 genes were expressed at higher levels in the model group compared with the control group, of which 1,011 overlapped with the 1,369 genes downregulated by *S. barbata* treatment. Conversely, 898 genes were expressed at lower levels in the model group than in the control group, with 896 overlapping the 1,715 genes upregulated by *S. barbata* (Figure 5B). Therefore, we focused on genes that exhibited consistent expression trends in both the control and *S. barbata* groups (either up- or downregulated compared to the model group) for subsequent enrichment and network analyses.

**FIGURE 5:**
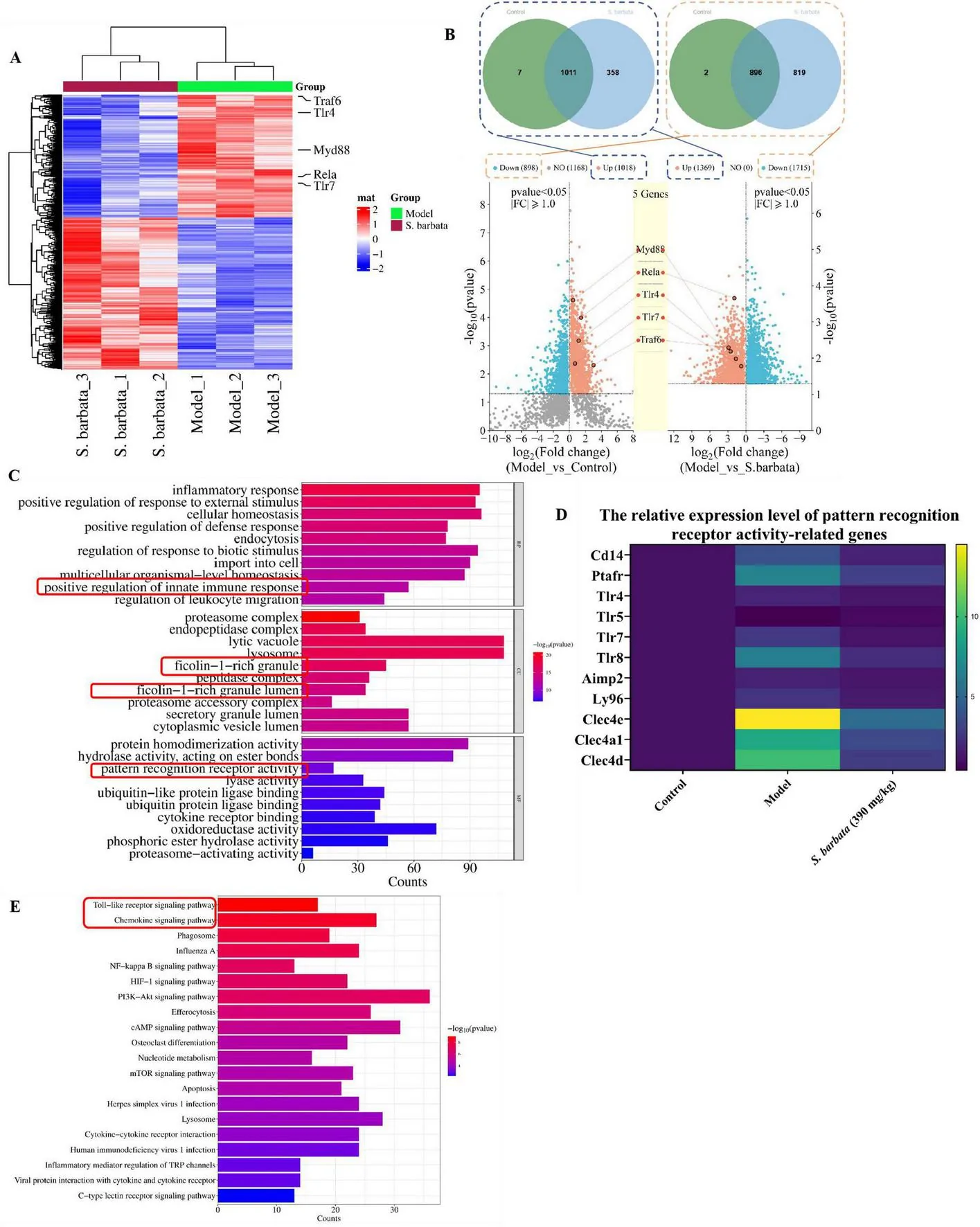
Differential gene expression analysis. Transcriptomic differential gene expression heatmap **(A)**; differential gene expression in Control group and *S. barbata* group compared to the Model group **(B)**; GO enrichment analysis **(C)**; the relative expression level of pattern recognitionreceptor activity-related gene **(D)**; KEGG enrichment analysis **(E)**.

Functional enrichment of the overlapping DEGs was performed using the Metascape database for Gene Ontology (GO) and Kyoto Encyclopedia of Genes and Genomes (KEGG) pathway analyses. A total of 304 GO terms were identified, including 177 biological process (BP) terms, 86 cellular component (CC) terms, and 71 molecular function (MF) terms (Figure 5C). The top 10 significantly enriched terms (ranked by *P*-value) were visualized. The enriched BP terms were mainly associated with inflammatory responses, positive regulation of response to external stimuli, defense response, positive regulation of innate immune response, and leukocyte migration, suggesting that *S. barbata* primarily modulates inflammatory and innate immune processes during H1N1 infection.

Following IAV infection, the earliest immune responses occur in airway and alveolar epithelial cells, where viral RNA activates toll-like receptors (TLRs), leading to the transcriptional induction of key signaling molecules and activation of interferon regulatory factors (IRFs). This process enhances the transcription of type I interferons (IFN-α/β) and activates the NF-κB signaling pathway, which induces the expression of proinflammatory cytokines and chemokines. The coordinated release of type I interferons, cytokines, and activation of cytotoxic T cells is critical for viral clearance.

At the molecular function level, enrichment was observed in pattern recognition receptor activity, lyase activity, and ubiquitin-like protein ligase binding. Among these, TLR4, TLR7, TLR8, PTAFR, CD14, and AIMP2–genes associated with pattern recognition receptor activity–were downregulated in the *S. barbata*-treated group compared with the model group, but showed higher expression relative to the control group (Figure 5D). This pattern suggests that *S. barbata* may exert its therapeutic effects by attenuating excessive activation of pattern recognition receptor–mediated innate immune responses during H1N1 infection.

Kyoto Encyclopedia of Genes and Genomes pathway enrichment of the common DEGs revealed that *S. barbata* significantly modulated several signaling pathways, with the toll-like receptor signaling pathway and cytokine–cytokine receptor interaction being the most prominently enriched (Figure 5E).

### The regulatory effect of *S. barbata* on the chemokine signaling pathway

To validate the RNA-seq results, the relative mRNA levels of 16 chemokine-related genes in mouse lung tissues were determined by qRT-PCR. As shown in Figure 6, infection with H1N1 markedly upregulated the expression of CCL11, CCL12, CCL2, CCL3, CCL4, CCL6, CCL7, CCL8, CCR1, CCR5, CXCL1, CXCL13, CXCL16, CXCL2, CXCL3, and CXCL9 in the model group, whereas these increases were reversed by *S. barbata* treatment (Figures 6A–P). The qRT-PCR data were highly consistent with the transcriptomic results, confirming both the inhibitory effect of *S. barbata* on chemokine signaling and the reliability of our RNA-seq findings.

**FIGURE 6:**
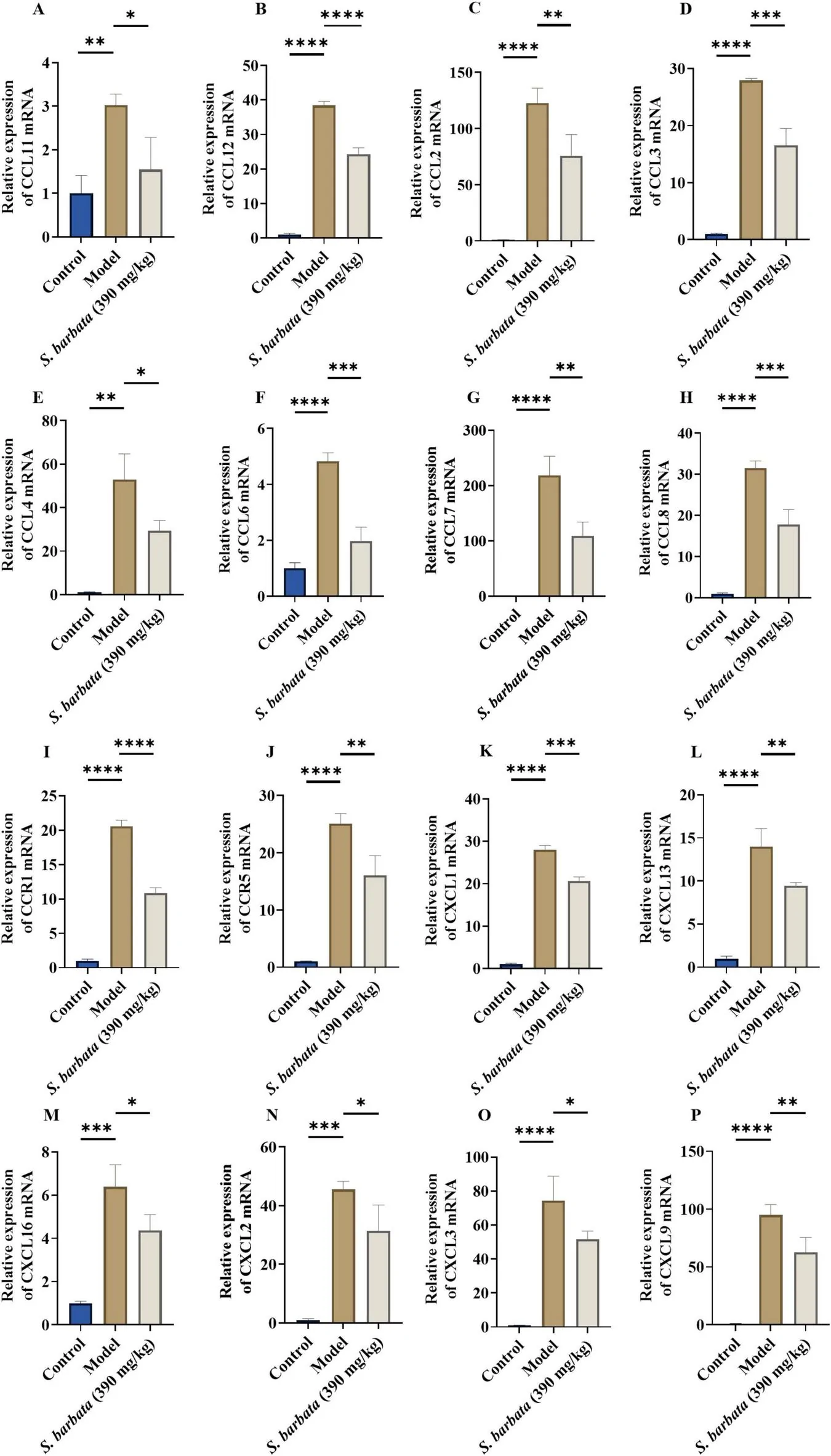
Expression of CCL11, CCL12, CCL2, CCL3, CCL4, CCL6, CCL7, CCL8, CCR1, CCR5, CXCL1, CXCL13, CXCL16, CXCL2, CXCL3, and CXCL9 mRNA in lung tissue (*n* = 6) **(A–P)**. *P < 0.05, **P < 0.01, ***P < 0.001, and ****P < 0.0001 indicate significant differences compared with the model group.

### The regulatory effect of *S. barbata* on the toll-like receptor signaling pathway

Furthermore, qRT-PCR analyses were conducted to examine key genes within the TLR signaling pathway. On day 6 post-infection, the mRNA levels of TLR4, TLR7, TLR8, Myd88, Traf6, RelA, IL-6, CD14, and CD80 were significantly elevated in the model group compared with controls, whereas *S. barbata* treatment markedly restored their expression toward baseline levels (Figures 7A–I). Western blot and immunohistochemical analyses were conducted to evaluate the expression of key proteins involved in the TLR signaling pathway. Immunohistochemical staining demonstrated that the elevated expression of RelA (Figure 8A) and TLR4 (Figure 8B) observed in the lung tissues of model mice was markedly attenuated following *S. barbata* treatment. Consistently, western blot results further confirmed that *S. barbata* significantly reduced the protein expression levels of TLR4, p-p65, p65, MyD88, and IκBα (Figures 8C–H).

**FIGURE 7:**
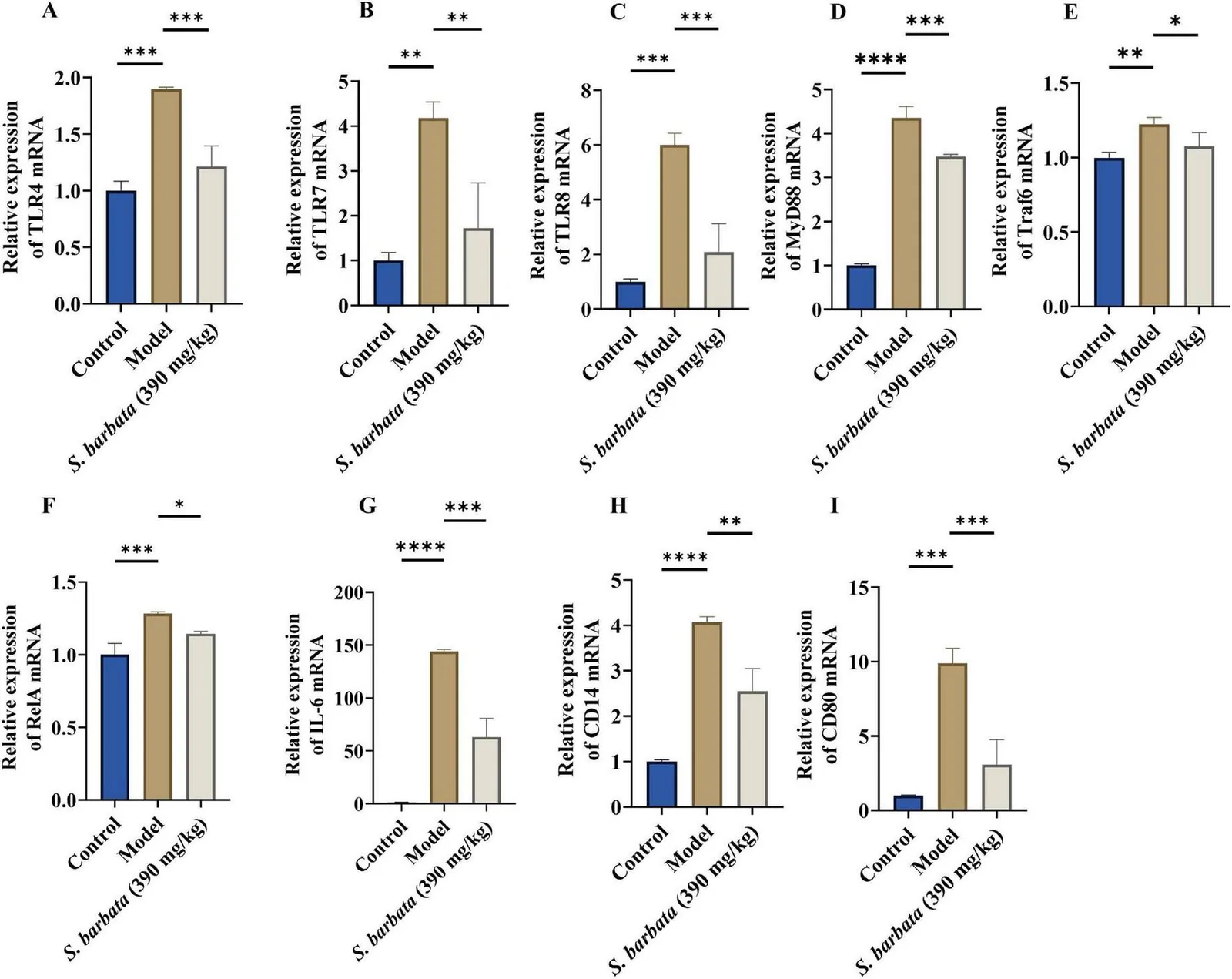
mRNA and protein expression of the TLR7 signaling pathway in lung tissue. Expression of TLR4, TLR7, TLR8, Myd88, Traf6, RelA, IL-6, CD14 and CD80 mRNA in lung tissue (*n* = 6) **(A–I)**. *P < 0.05, **P < 0.01, ***P < 0.001, and ****P < 0.0001 indicate significant differences compared with the model group.

**FIGURE 8:**
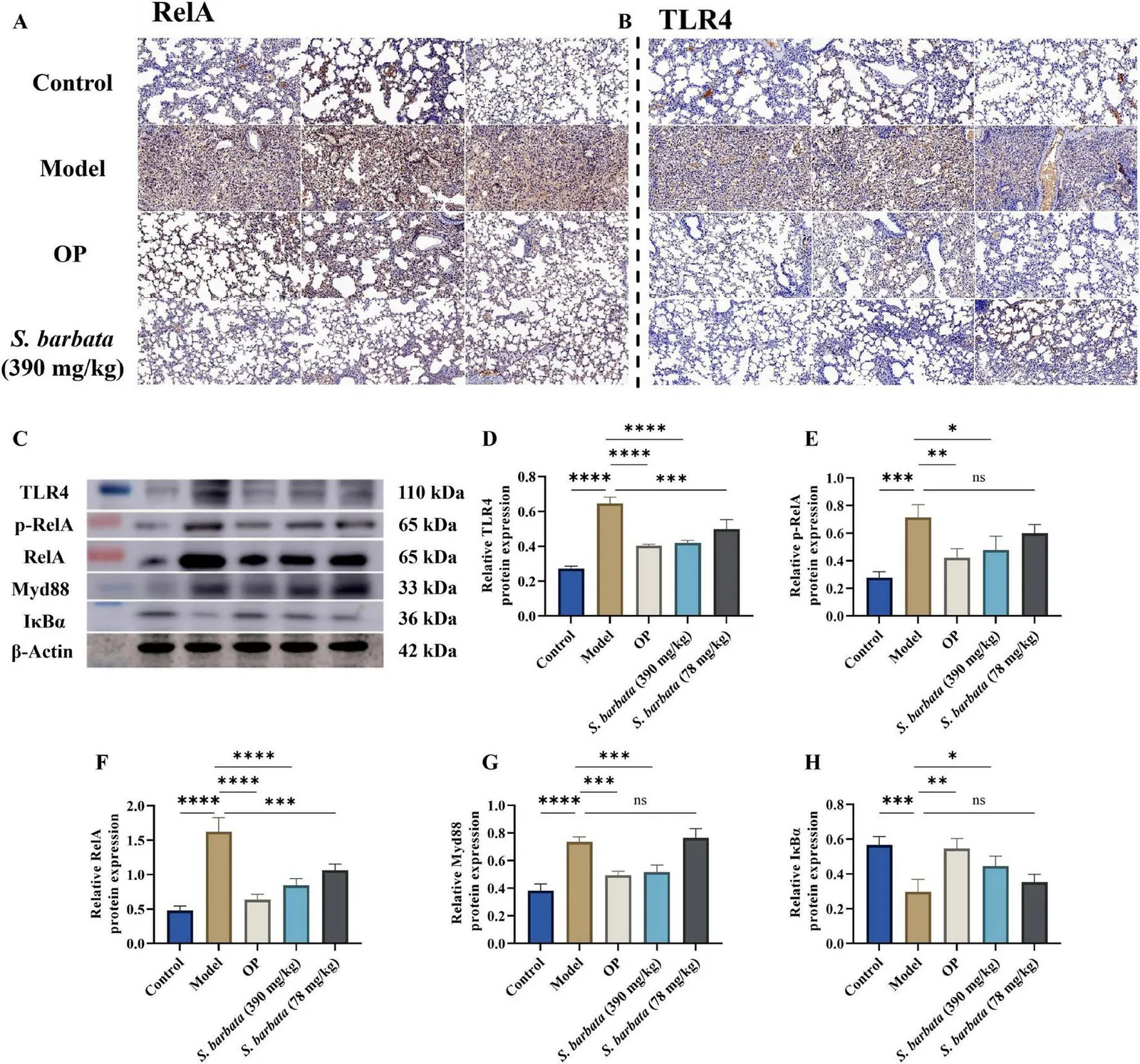
Immunohistochemical analysis of RelA **(A)** and TLR4 **(B)** protein expression in lung tissues (*n* = 3). Representative western blot images **(C)**. Quantitative analysis data of TLR4 **(D)**, p-Rela **(E)**, Rela **(F)**, Myd88 **(G)**, IκB-α **(H)** (*n* = 3). *P < 0.05, **P < 0.01, ***P < 0.001, and ****P < 0.0001 indicate significant differences compared with the model group; ns indicates no significant difference.

### Identification of bioactive constituents

(1) Blood-absorbed components

The total ion chromatograms (TICs) of the prototype compounds of *S. barbata*, blank serum, and serum samples containing absorbed *S. barbata* constituents in both negative and positive ion modes are presented in Figures 9A–F. A total of 122 prototype compounds from *S. barbata* were detected in mouse plasma. These included 17 phenylpropanoids, 3 pyridines and derivatives, 7 phenols, 13 terpenes, 2 quinones, 27 flavonoids, 16 carbohydrates and glycosides, 2 steroids, 8 fatty acyls, 3 organic acids and derivatives, 2 carboxylic acids and derivatives, 4 lipids and lipid-like molecules, 9 organoheterocyclic compounds, 1 indole derivative, and 8 other minor compounds (Figure 9G and Supplementary Table 1).

**FIGURE 9:**
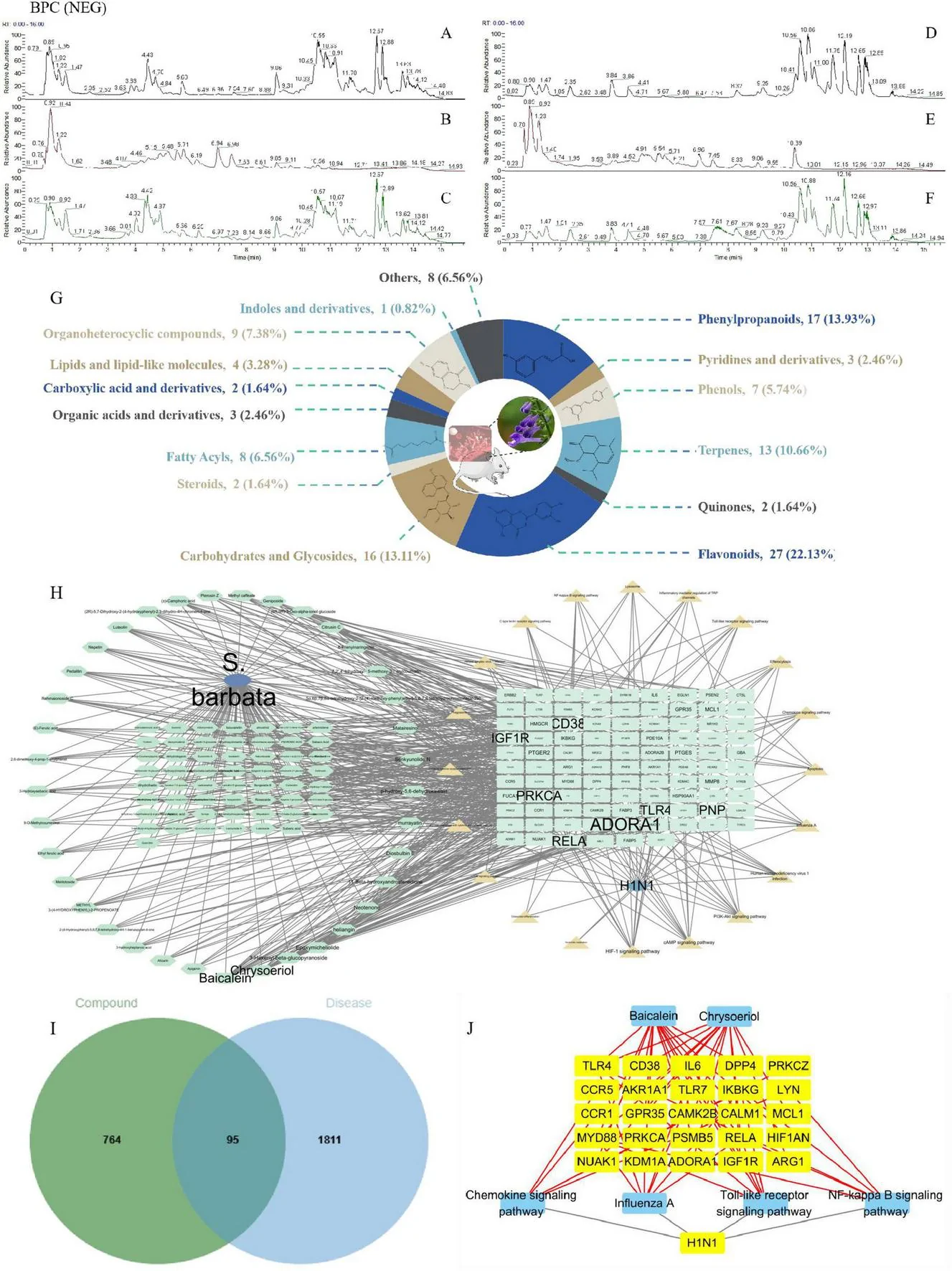
Identification and network analysis of blood-absorbed constituents of *S. barbata*. Total ion chromatograms (TICs) of *S. barbata* prototype compounds **(A)**, mouse serum constituents **(B)**, and *S. barbata*-treated mouse serum constituents in negative ion mode **(C)**, TICs of *S. barbata* prototype compounds **(D)**, mouse serum constituents **(E)**, and *S. barbata*-treated mouse serum constituents in positive ion mode **(F)**, classification of blood-absorbed constituents of *S. barbata* **(G)**, network analysis of active compounds **(H)**, Venn diagram of overlapping compound–disease targets **(I)**, H1N1-related pathways potentially regulated by baicalein and chrysoeriol **(J)**.

(2) Network pharmacology analysis

The potential targets of the absorbed compounds were retrieved from public databases, yielding 859 predicted targets for 105 compounds. These were intersected with the 1,907 DEGs that exhibited the same regulatory trends between the treatment/control groups and the model group, resulting in 94 compounds corresponding to 96 overlapping targets (Figure 9I). KEGG pathway enrichment analysis of these overlapping targets was then performed to elucidate the biological pathways involved. A “compound–target–pathway–disease” network was constructed and visualized using Cytoscape 3.6.0. The size of each node (compound, target, or pathway) was proportional to its degree value. In the visualization, ellipses represent *S. barbata*, hexagons represent compounds, squares denote targets, triangles indicate pathways, and diamonds represent the H1N1 disease node (Figure 9H).

Among all compounds, baicalein (degree = 15) and chrysoeriol (degree = 14) exhibited the highest connectivity, suggesting strong multitarget interactions. Further analysis revealed that both compounds were associated with 25 key targets–including TLR7, TLR4, and RELA–and were enriched in pathways directly related to influenza A infection, chemokine signaling, and the toll-like receptor–NF-κB cascade. These findings imply that baicalein and chrysoeriol may serve as principal active compounds mediating the anti-H1N1 effects of *S. barbata* through the regulation of innate immune and inflammatory pathways (Figure 9J).

### Validation of anti-H1N1 activities of baicalein and chrysoeriol

The CC_50_ values of both chrysoeriol and baicalein in MDCK cells exceeded 500 μM, whereas their IC_50_ values against H1N1 were 11.10 μM (Figure 10A) and 13.80 μM (Figure 10B), respectively. In addition, oseltamivir phosphate (OP), used as a positive control, exhibited an IC_50_ of 4.45 μM against H1N1 (Figure 10C). These findings indicate that chrysoeriol and baicalein exhibit potent antiviral activity against H1N1 within a safe concentration range in vitro. *In vivo* experiments further confirmed these effects. Treatment with baicalein and chrysoeriol mitigated H1N1-induced body weight loss (Figure 10D), significantly reduced the lung index (Figure 10E), and increased the spleen index (Figure 10F), though no significant change was observed in the thymus index (Figure 10G). Both compounds markedly decreased viral loads in lung tissues (Figure 10H) and alleviated histopathological signs of pulmonary inflammation (Figure 10I). Collectively, these results demonstrate that baicalein and chrysoeriol possess strong *in vitro* and *in vivo* anti-H1N1 efficacy, supporting their role as major bioactive constituents of *S. barbata* responsible for its antiviral effects.

**FIGURE 10:**
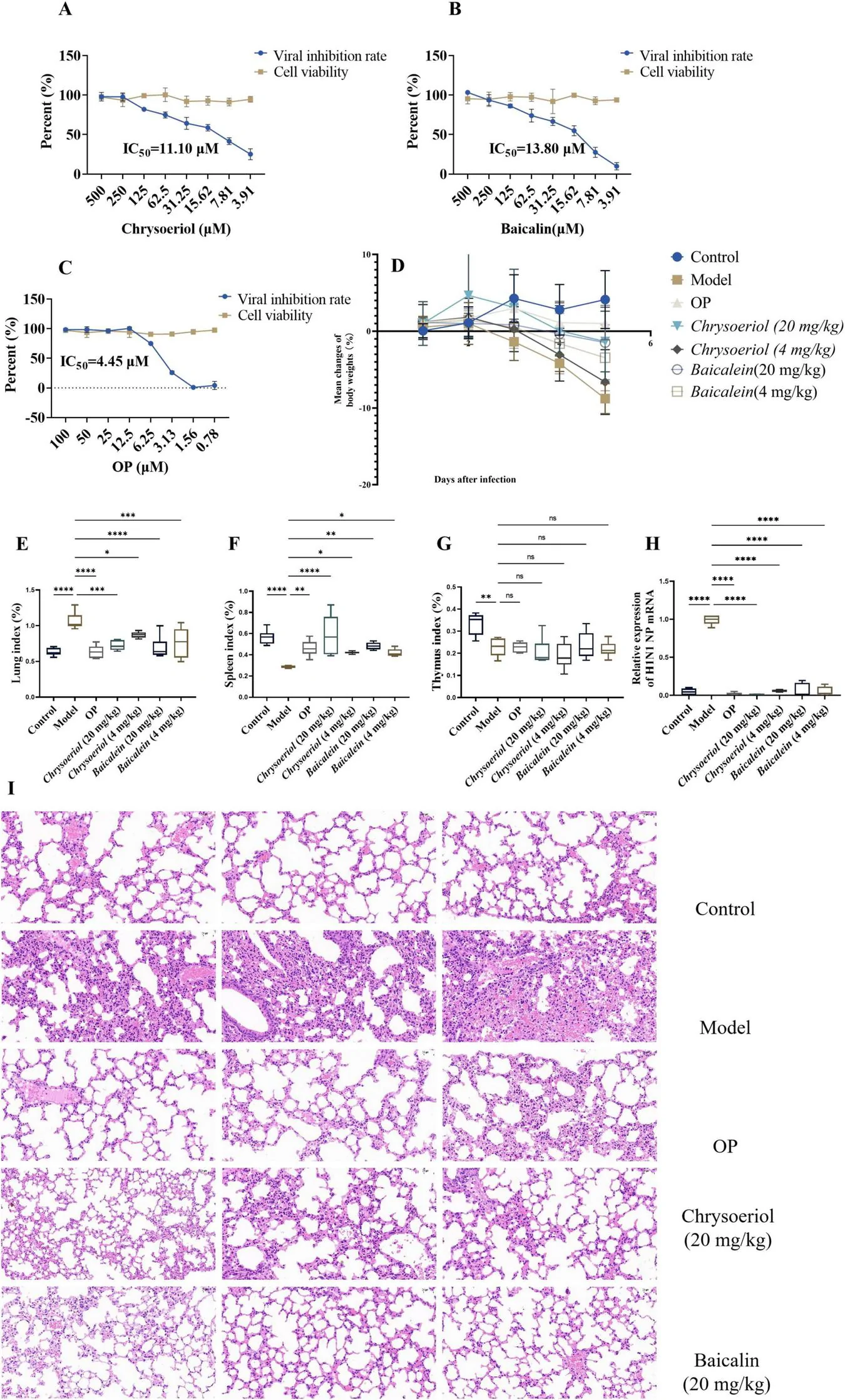
Validation of the antiviral activity of baicalein and chrysoeriol. *In vitro* cell viability curves of chrysoeriol **(A)**, baicalein **(B)**, and OP **(C)**, *in vivo* activity assays showing body weight changes **(D)**, lung indices **(E)**, spleen indices **(F)**, and thymus indices **(G)**, lung viral load **(H)**, and H&E-stained lung tissue sections in mice **(I)**. *P < 0.05, **P < 0.01, ***P < 0.001, and ****P < 0.0001 indicate significant differences compared with the model group; ns indicates no significant difference.

## Discussion

### Direct and indirect anti-H1N1 effects of *S. barbata*

The antiviral activity of *S. barbata* extract against H1N1 infection was first evaluated in an MDCK cell model. The extract exhibited a IC_50_ of 42.87 μg/mL against H1N1, while showing negligible cytotoxicity toward MDCK cells (CC_50_ > 500 μg/mL). Within the non-cytotoxic concentration range (100 and 200 μg/mL), *S. barbata* treatment markedly reduced viral titers in the supernatant and decreased the intracellular expression level of the viral NP. Consistent with the *in vitro* findings, *S. barbata* also demonstrated significant protective efficacy against H1N1 infection *in vivo*. Direct antiviral effects are generally defined as interference with viral proliferation processes, including adsorption, penetration, replication, assembly, or release, or by damaging viral structural proteins. HA and NA are characteristic surface glycoproteins of H1N1, while NP and matrix protein M2 serve as core structural proteins. RT-qPCR analysis revealed that *S. barbata* significantly downregulated the mRNA expression of HA, NA, NP, and M2 in mouse lung tissues, showing a consistent inhibitory trend across all four genes. These results suggest that *S. barbata* exerts a direct inhibitory effect on H1N1 viral replication.

In addition to direct antiviral action, *S. barbata* also exerts indirect effects by alleviating virus-induced tissue injury and modulating host immune responses. Following H1N1 invasion, infected mice typically exhibit pulmonary congestion, edema, and increased lung mass. The lung index serves as a key indicator of pulmonary inflammation and is widely used to evaluate the anti-H1N1 efficacy of candidate drugs. Moreover, the developmental state of immune organs such as the spleen and thymus reflects the overall immune competence of the host. H1N1 infection generally reduces the spleen and thymus indices due to excessive immune cell consumption. *S. barbata* administration significantly decreased the lung index while restoring the spleen and thymus indices, indicating that the extract protects the host through immunoregulatory mechanisms.

Upon influenza virus infection, the host immune system releases a variety of cytokines to inhibit viral replication and regulate immunity. However, excessive cytokine secretion can trigger uncontrolled inflammation and tissue injury, a phenomenon often referred to as a “cytokine storm.” Thus, suppressing hyperinflammatory cytokine release is critical for mitigating H1N1-induced lung damage. Overexpression of IL-1β promotes excessive recruitment and activation of neutrophils and macrophages, while IL-6 facilitates neutrophil chemotaxis and local infiltration. TNF-α induces leukocyte migration and promotes degranulation of granulocytes, amplifying the inflammatory cascade. Elevated levels of IL-1β, IFN-γ, IL-6, and TNF-α have been correlated with the severity of H1N1 infection in clinical cases. In our study, *S. barbata* significantly reduced the levels of these pro-inflammatory cytokines in lung homogenates, consistent with histopathological findings showing alleviated pulmonary injury. IL-2 is a key cytokine that promotes the differentiation of CD8^+^ T cells into cytotoxic T lymphocytes during viral infection (Zhou et al., 2021). Our results showed that *S. barbata* treatment significantly decreased IL-2 levels in the lungs of H1N1-infected mice. Therefore, we further explored its regulatory effects on CD4^+^ and CD8^+^ T cell populations. CD8^+^ T cells play a direct role in killing infected cells, whereas CD4^+^ T cells enhance the effector functions of innate immune cells and B cells. An imbalance in the CD4^+^/CD8^+^ ratio has been associated with increased severity of H1N1-induced pneumonia. Flow cytometry revealed that *S. barbata* decreased the proportion of CD4^+^ T cells in the spleen without significantly affecting CD8^+^ T cells, suggesting that the extract may suppress overactivation of CD4^+^ T cell-mediated immune responses. Moreover, *S. barbata* partially restored the Th1/Th2 balance by increasing both Th1 and Th2 subpopulations, indicating a bidirectional regulatory effect. Interestingly, despite the overall increase in CD4^+^ T cell proportion in the model group, both Th1 and Th2 subsets were reduced, implying expansion of other CD4^+^ subpopulations–a phenomenon warranting further investigation.

### Molecular mechanisms underlying the anti-H1N1 effects of *S. barbata*

Transcriptomic analysis provided deeper insight into the molecular basis of *S. barbata*’s anti-H1N1 activity. GO enrichment analysis indicated that the DEGs after *S. barbata* treatment were mainly involved in biological processes such as regulation of inflammatory responses, positive regulation of innate immunity, and leukocyte migration. The enriched cellular components included “ficolin-1-rich granule” and “ficolin-1-rich granule lumen.” Ficolin-1-rich granules store and release Ficolin-1 upon stimulation; Ficolin-1 functions as a soluble pattern recognition receptor that recognizes pathogen-associated molecular patterns (PAMPs) and activates the complement system via the lectin pathway, thereby participating in innate immune signaling.

Kyoto Encyclopedia of Genes and Genomes pathway enrichment further revealed that the toll-like receptor (TLR) signaling pathway was the most significantly modulated pathway following *S. barbata* treatment. During IAV infection, viral RNA within airway and alveolar epithelial cells activates TLRs, leading to the induction of interferon regulatory factors and type I interferons (IFN-α/β). This activation can subsequently trigger the NF-κB pathway, promoting the expression of proinflammatory cytokines and chemokines. While these responses are essential for viral clearance, excessive activation results in tissue injury. Our data indicate that *S. barbata* alleviates H1N1-induced pathology by suppressing the overactivation of the TLR and NF-κB pathways. These findings were further validated by RT-qPCR and immunohistochemical analyses, which confirmed the downregulation of key genes in the TLR and chemokine signaling pathways.

### Identification and validation of the active compounds responsible for the anti-H1N1 activity

Following oral administration, most herbal components undergo absorption, distribution, and metabolism before reaching their target tissues. Pharmacological activity is generally mediated by prototype or metabolized compounds that enter systemic circulation. Using UPLC-MS, we identified 122 blood-absorbed constituents of *S. barbata*. By integrating network pharmacology and transcriptomic analyses, we intersected the predicted targets of these compounds with DEGs identified in the transcriptomic data, constructing a “*S. barbata*–compound–target–pathway–H1N1” interaction network. Among these, baicalein and chrysoeriol exhibited the highest degree values in the network, indicating their central regulatory roles. These two flavonoids were associated with multiple antiviral and immune-related pathways, including the influenza A pathway, chemokine signaling pathway, toll-like receptor signaling pathway, and downstream NF-κB pathway. Subsequent *in vitro* and *in vivo* experiments confirmed that both baicalein and chrysoeriol possessed potent inhibitory effects against H1N1 infection, validating the network-based predictions.

## Data Availability

The data presented in this study are deposited in the NCBI Sequence Read Archive (SRA), accession number PRJNA1522933.
